# Arthralgia in female Masters weightlifters

**DOI:** 10.1186/s12891-023-06814-y

**Published:** 2023-08-24

**Authors:** Marianne Huebner, Mark E. Lavallee

**Affiliations:** 1 Department of Statistics and Probability, Michigan State University, East Lansing, MI USA; 2 Department of Kinesiology, Michigan State University, East Lansing, MI USA; 3 Department of Orthopedics, UPMC Central Pennsylvania, Harrisburg, PA USA; 4 USA Weightlifting Sports Medicine Society, Colorado Springs, CO USA; 5 Executive Medical Committee, International Weightlifting Federation, Lausanne, Switzerland

**Keywords:** Olympic-style weightlifting, Women, Aging, Joint pain, Menopause

## Abstract

**Background:**

Arthralgia or joint pain is a heterogeneous condition including organic and nonorganic joint pain. It is common in older populations, particularly in females. There is evidence that menopausal changes are associated with increased prevalence of arthralgia. While physical activities have been recommended to mitigate osteoarthritis (OA) and arthralgia, sport participation also carries risk factors due to excessive loading of some joints and possible injuries. The aim was to evaluate the association of training patterns, prior injuries, and severity of menopausal symptoms with arthralgia in female Masters weightlifters.

**Methods:**

Competitive female Masters weightlifters (*n=*868, 30-78 years) from 30 countries completed an online survey including joint pain for different anatomical sites, weightlifting training and performance, sport history, and menopausal symptoms. Logistic regression models were used to estimate the association of training patterns, prior sport participation, and menopausal symptom severity with arthralgia separately for shoulders, spine, hips, knees, ankles, elbows, and hands.

**Results:**

Arthralgia was most reported in knees (38.8%), shoulders (29.8%), hands/wrists (28.8%), and hips (24.9%). The prevalence of arthralgia was 51.5% in pre-menopausal women, 62.4% in women post natural menopause and 73.3% in women post medical or surgical menopause. Lifting heavier weights was associated with arthralgia in hips (OR=1.05, *p=*0.03), knees (OR=1.06, *p=*0.01), and hands/wrists (OR=1.05, *p=*0.04), but prior strength training was protective for arthralgia in the shoulders (OR=0.66, *p=*0.02). Prior injuries and psychological menopausal symptom severity were associated with an increased risk for arthralgia (*p<*0.01).

**Conclusions:**

Arthralgia was common in competitive female weightlifters. Training frequency was not associated with arthralgia, but lifting heavier weights relative to age and body mass was. Prior injuries and menopausal symptoms were associated with arthralgia, but prior strength training was protective of arthralgia in the shoulders. Athletes, coaches and sports medicine professionals should be aware that prevalence of polyarthralgia increases in post-menopausal athletes.

## Background

Arthralgia or joint pain is a heterogeneous condition and is inclusive of osteoarthritis (OA), degenerative joint disease, and organic and nonorganic causes of joint pain. Its prevalence may be underestimated since not all symptomatic individuals seek medical help for diagnosis [1]. It is a common condition among older populations and can lead to reduced quality of life and impaired function [2]. For example, joint pain or backache has been reported in over 50% of women in cross-sectional studies [3]. There is no single diagnosis for arthralgia, and many studies focused only on osteoarthritis. However, risk factors for arthralgia may differ from those identified for osteoarthritis [4, 5].

Physical activities have been recommended for improving OA to increase strength, joint stability, proprioception, flexibility, and reduce pain [6–8]. Sport participation provides other health benefits, including bone health, cardiovascular function, and quality of life [2]. However, recreational activities and prior injuries have been shown to be associated with OA [9], and athletes are more likely to sustain joint injuries compared to other individuals. Depending on the type of sport certain joints may be more likely to be affected due to excessive loading of those joints [2, 6, 10]. For example, handball, ice hockey, and wrestling were associated with a greater risk of developing hip OA [2]. Competitive Masters weightlifters (defined as lifters 30 years or older) may have a higher risk for developing arthralgia in some joints. Common anatomical sites for injuries in Masters weightlifters were shoulders, back, knees, and wrists [11], but arthralgia has not been studied in weightlifters.

Arthralgia and OA increases with age and are more common in women [12, 13]. Menopausal status and depressed mood have been shown to be associated with arthralgia which could be related to the reduction in circulating estrogen levels [3].

There is a significant gap in the literature investigating arthralgia in female athletes, in particular its association with menopausal status or menopausal symptom severity. The objectives of this study were to evaluate prevalence and determinants for arthralgia for different anatomical sites in competitive female Masters weightlifters. We expected that prevalence of arthralgia would be more common in shoulders, knees, and wrists compared to other joints due to the demands of this sport. Our hypothesis was that training patterns (frequency and load), sport history, and severity of menopausal psychological symptoms would be associated with this condition.

## Methods

### Design and Participants

A cross-sectional study design was employed. An online survey was completed by 912 female weightlifters, ages 30 to 79, from 30 countries. The survey was distributed by the International Masters Weightlifting Association (IMWLA) to the National Masters Chairs. They then used email or social media to widely communicate the study to the female members in their organization. In addition, the survey was advertised in Olympic-style weightlifting interest groups via Facebook and Instagram. The survey was available in four languages (English, German, French, Spanish), translated and tested by native speakers. Data collection was conducted in Spring 2022. The study protocol was reviewed and determined to be exempt by the Michigan State University Human Research Ethics Committee (STUDY00007512). Inclusion criteria were female Masters weightlifters, globally. The Masters age groups start at 35 years in international competitions, but in some countries, the Masters age also encompasses 30 to 34 years, hence this study included female weightlifters ages 30 and older. Exclusion criteria were younger than 30 years (*n=*1) or currently pregnant (*n=*3). To account for the possibility of male participants, missing responses to age of menstruation or prior pregnancies were excluded (*n=*22). Since the focus was on active Olympic-style weightlifters, missing best snatch or clean and jerk in the last 6 months (*n=*18) was also an exclusion criterion. This resulted in an analysis data set of 868 females [14].

### Measures

Arthralgia is a multifactorial condition with a lack of standardized definition [15]. Arthralgia at different anatomical sites (shoulders, knees, hips, spine, hands/wrists, elbows, or ankles) was defined by a positive answer to the questions *“Have you experienced any pain or swelling in any of your joints at any time in the past six months?”* or *“Have you visited any of the following health professionals in the past 6 months because of problems with your bones, joints or muscles?”* The age of first occurrence of joint pain was indicated by responses to *“Has a doctor or other health professional told you that you have any joint problems, rheumatism or arthritis? If yes, at what age?”* and *“At what age did the pain or swelling in any of your joints occur for the first time?”* Since prior injuries may be associated with arthralgia a question about occurrence for the specific joints and age was included *“Have you had a prior injury in any of the joints?”* and *“At what age were you injured for the first time in these joints?”*

Variables related to weightlifting activity were hours of weekly weightlifting training and best snatch and best clean & jerk performance in the last 6 months. While best snatch and best clean & jerk lifts may not have been achieved in the same training session or competition, the weights lifted were summed and adjusted with Sinclair-Huebner-Meltzer-Faber points (SHMF) to represent the performance level. SHMF standardizes performances with respect to body mass and age [16]. SHMF was used as a proxy for training load, since a maximum weight can be achieved by lifting heavier during training sessions [17]. Variables related to sport history were prior participation in high impact sports (e.g., ball sports, gymnastics, martial arts), prior participation in strength sports (e.g., powerlifting or body building), age of first competition in any sport and age at start of weightlifting.

The Menopausal Rating Scale (MRS) [18] was used to calculate menopausal symptom severity. Analysis was based on the score for the psychological subscale (depressive mood, irritability, anxiety, and physical and mental exhaustion).

### Statistical analysis

Continuous variables were summarized with mean ± standard deviation, categorical variables with counts and percentages. Proportions of females with arthralgia prior and post menopause were compared with Fisher’s exact test. Logistic regression models were used to estimate the effect of age, training hours, training load (with SHMF as a proxy for weights lifted, adjusted for age and body mass), and severity of menopausal symptoms on arthralgia. The psychological subscale of the menopausal symptom rating scale was included, since depressed mood had been shown previously to be associated with arthralgia [19]. The somato-vegetative subscale was not included in the model, since joint pain is one of the items. Other covariates were BMI and prior participation in high impact sports which are known risk factors for osteoarthritis [20]. Competition experience prior to age 20 was included in the model due to studies of osteoarthritis in former athletes [21]. We tested the association of these variables with arthralgia in specific anatomical sites (shoulders, spine, hips, knees, elbows, hand/wrists, ankles). All analyses were conducted with the statistical software R version 4.3.0 [22]. *P*-values less than 0.05 were considered statistically significant. The study was reported according to the STROBE statement [23].

## Results

The mean age of the respondents was 43.6 years. The majority started weightlifting before age 35 (51.9%), but 11.2% indicated starting weightlifting after age 50 (Table 1). The majority had competed in some sport prior to age 20 (55.9%), but only 4.5% trained in weightlifting before age 20. The mean number of hours of weightlifting each week was seven hours.

**Table 1:** Characteristics of participants

	N	Descriptive statistics^a^
Age	868	43.6 ± 9.8
Age at starting WL	867	
<35		51.9 %, 450
35-49		36.9 %, 320
50+		11.2 %, 97
Height, cm	847	166.0 ± 55.2
Weight, kg	861	71.7 ± 15.1
BMI	843	26.6 ± 5.1
Competing before age 20 (all sports)	868	55.9 %, 485
Start weightlifting before age 20		4.5%, 39
Training, hours/week	863	7.0 ± 3.0
Any arthralgia in the last 6 months	868	49.7%, 435
Visit to a health professional in the last 6 months for problems with bones, joints, muscles		
Any health professional	841	73.8%, 621
General practitioner/ Family doctor/PCP	828	26.7%, 221
Specialist physician	825	29.0 %, 239
Hospital	808	5.3%, 43
Physiotherapist	820	35.6%, 292
Occupational therapist	809	3.3%, 27
Naturopath	805	4.8 %, 39
Acupuncturist	810	13.2 %, 107
Chiropractor	819	39.3 %, 322

^a^Continuous variables are summarized with mean ± standard deviation, categorical variables with percentages and counts

Almost half of respondents experienced arthralgia in the past 6 months (49.7%), and 73.8% visited health professionals in the last six months for problems with bones, joints, or muscles. Joint pain was mostly reported in knees (38.8%) followed by shoulders (29.8%), hands/wrists (28.8%), and hips (24.9%) (Table 2). The age of first occurrence of joint pain was in their thirties, while prior injuries first occurred in their mid-twenties except ankle injuries which first occurred at an average age of 16. Most common location of prior injuries were at knees (37.4%), shoulders (39.2%), ankles (31.8%), and hands/wrists (28.9%).

**Table 2:** Prevalence of arthralgia in the past six months and prevalence of prior injuries from any activities stratified by joints in 868 participants

	**N**	**Arthralgia past 6 months , % (95% CI)**	**Age at first arthralgia occurrence (median, quartiles)**	**N**	**Prior injury, % (95% CI)**	**Age at first injury occurrence (median, quartiles)**
Shoulder	259	29.8 (26.8, 33.0)	36 (29, 45)	341	39.2 (36.0,42.6)	33 (25, 42)
Spine	138	15.9 (13.5, 18.5)	32 (25, 41)	172	19.8 (17.2, 22.6)	26 (16, 34)
Hips	216	24.9 (22.1,27.9)	30 (30, 45)	143	16.4 (14.1, 19.1)	26 (16, 40)
Knees	337	38.8 (35.6, 42.1)	33 (25, 42)	335	37.4 (34.2, 40.7)	23 (16, 35)
Elbows	122	14.1 (11.8,16.6)	40 (30, 46)	102	11.7 (9.7, 14.1)	32 (16, 41)
Ankles	89	10.3 (8.3,12.5)	32 (16, 45)	276	31.8 (28.7, 35.0)	16 (12, 27)
Hands/wrists	248	28.6 (25.6,31.7)	38 (30, 46)	251	28.9 (25.9, 32.1)	28 (13, 36)
Physician diagnosis of any arthralgia (*n=*835)	163	19.5 (16.9, 22.4)	38 (30, 48)			

*Abbreviations*: *CI* Confidence interval

Severity of psychological menopausal symptoms and prior injuries were associated with arthralgia (Table 3). Prior participation in high impact sports (ball sports, martial arts, gymnastics) and competing prior to age 20 were not significantly associated with arthralgia at any of the joints (shoulders, hips, knees, spine, elbows, hands/wrists, ankles) (Table 3). Hours of weekly training was not associated with arthralgia, but maximum weight lifted as measured by SHMF was a risk factor for arthralgia overall (OR=1.01, *p=*0.01) and in hips (OR=1.05, *p=*0.03), knees (OR=1.06, *p=*0.01), elbows (OR=1.08, *p=*0.005), and hands/wrists (OR=1.05, *p=*0.04). Prior participation in strength sports (body building, power lifting) was protective of arthralgia in shoulders (OR=0.69, *p=*0.04). Older age was associated with arthralgia overall (OR=1.03, *p<*0.01), in knees (OR=1.01, *p=*0.04) and hands/wrists (OR=1.01, *p=*0.04).

**Table 3:** Odds ratios and 95% confidence intervals for arthralgia in the last 6 months

	**Overall**	**Shoulders**	**Hips**	**Knees**
	(*n=*803, events=437)	(*n=*822, events=242)	(*n=*822, events=203)	(*n=*822, events=322)
Age	1.03 (1.02, 1.05); *p<*0.01	1.01 (1.00, 1.03); *p=*0.09	1.02 (1.00, 1.03); *p=*0.06	1.01 (1.00, 1.03); *p=*0.04
BMI	1.05 (1.01, 1.08) *p<*0.01	1.03 (1.00, 1.06); *p=*0.07	1.04 (1.01, 1.07); *p=*0.03	1.05 (1.02, 1.09); *p<*0.01
Training, hours/week	1.01 (0.96,1.06) *p=*0.60	1.02 (0.97, 1.08); *p=*0.38	1.02 (0.96, 1.08); *p=*0.57	1.01 (0.96, 1.06); *p=*0.74
SHMF, 10 points	1.05 (1.01,1.10) *p=*0.01	1.02 (0.98,1.07) *p=*0.27	1.05 (1.01,1.10) *p=*0.03	1.06 (1.01,1.10) *p=*0.01
Prior impact sports	1.37 (0.92, 2.04); *p=*0.12	1.09 (0.71, 1.69); *p=*0.69	1.31 (0.81, 2.11); *p=*0.27	1.30 (0.85, 1.98); *p=*0.22
Prior strength sports	0.97 (0.70, 1.33); *p=*0.85	0.69 (0.49, 0.98); *p=*0.04	0.83 (0.58, 1.19); *p=*0.32	0.81 (0.58, 1.12); *p=*0.20
Competing prior to age 20	1.13 (0.83, 1.52); *p=*0.44	1.08 (0.78,1.50); *p=*0.64	1.10 (0.78, 1.55); *p=*0.59	1.26 (0.93, 1.73); *p=*0.13
Prior injury at joint		1.58 (1.15 2.16); *p<*0.01	2.19 (1.49, 3.22); *p<*0.01	2.23 (1.65, 3.00); *p<*0.01
MRS psychological symptoms	1.13 (1.08, 1.19); *p<*0.01	1.12 (1.07, 1.18); *p<*0.01	1.11 (1.05, 1.17); *p<*0.01	1.11 (1.06, 1.17); *p<*0.01
	**Spine**	**Elbows**	**Hands/Wrists**	**Ankles**
	(*n=*822, events=132)	(*n=*822, events=119)	(*n=*822, events=235)	(*n=*822, events=83)
Age	1.00 (0.98, 1.02); *p=*0.71	1.01 (0.99, 1.03); *p=*0.23	1.01 (1.00, 1.03); *p=*0.04	1.02 (0.99, 1.04); *p=*0.18
BMI	0.99 (0.95, 1.02); *p=*0.58	0.99 (0.95, 1.03); *p=*0.65	1.03 (1.01, 1.07); *p=*0.02	1.04 (1.00,1.09); *p=*0.06
Training, hours/week	0.99 (0.92, 1.06); *p=*0.69	0.99 (0.93, 1.06); *p=*0.84	1.01 (0.95, 1.07); *p=*0.77	1.00 (0.92,1.09); *p=*0.96
SHMF, 10 points	1.04 (0.98,1.10) *p=*0.16	1.08 (1.02, 1.15) *p=*0.005	1.05 (1.00, 1.10) *p=*0.04	0.98 (0.91,1.05) *p=*0.53
Prior impact sports	0.96 (0.55, 1.67); *p=*0.887	0.87 (0.51, 1.50); *p=*0.63	1.12 (0.72, 1.73); *p=*0.62	0.89 (0.46, 1.72); *p=*0.72
Prior strength sports	0.79 (0.51, 1.22); *p=*0.29	0.85 (0.54, 1.32); *p=*0.47	0.91 (0.64, 1.29); *p=*0.59	0.84 (0.49, 1.44); *p=*0.53
Competing prior to age 20	1.66 (1.09, 2.53); *p=*0.02	1.09 (0.72, 1.65); *p=*0.69	0.78 (0.56, 1.08); *p=*0.13	0.96 (0.58,1.59); *p=*0.88
Prior injury at joint	2.92 (1.93, 4.42); *p<*0.01	1.57 (0.93, 2.65); *p=*0.09	1.35 (0.96, 1.89); *p=*0.09	3.01 (1.84, 4.92); *p<*0.01
MRS psychological symptoms	1.12 (1.06, 1.19); *p<*0.01	1.11 (1.04, 1.18); *p<*0.01	1.15 (1.09, 1.21); *p<*0.01	1.21 (1.13, 1.30); *p<*0.01

*Abbreviations*: *BMI* Body mass index, *SHMF* Sinclair-Huebner-Meltzer-Faber points, *MRS* Menopausal Rating Scale

**Fig. 1:**
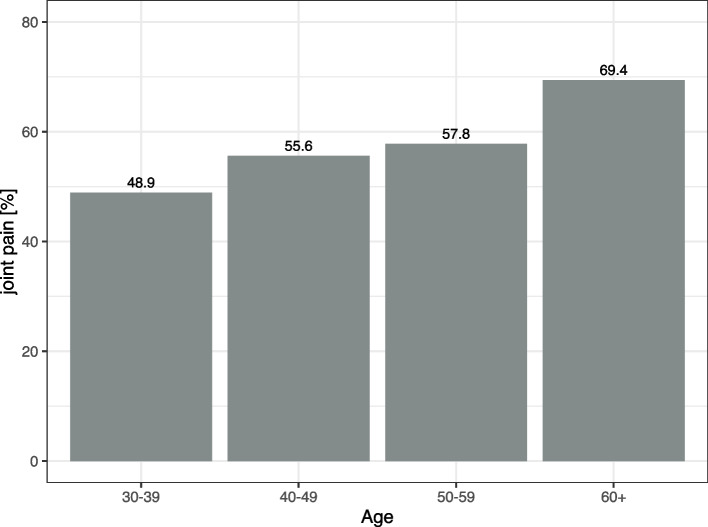
Proportion of respondents with arthralgia by decade of age

**Fig. 2:**
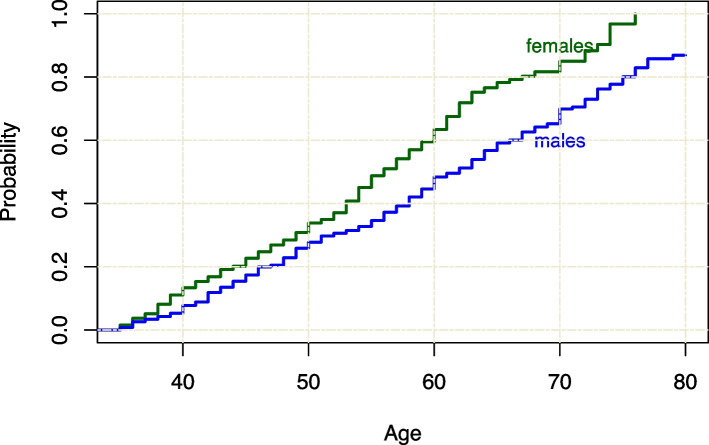
Cumulative incidence curve of polyarthralgia in a 2021 survey of male and female Master weightlifters (*p<*0.01)

Prevalence of arthralgia increased with age (Fig. 1) and was associated with menopausal status. The proportion of females with arthralgia was 51.5% in pre-menopause, 62.4% in natural menopause (*p=*0.03 compared to pre-menopause), and 73.3% in medical or surgical menopause (*p<*0.01 compared to pre-menopause). In a 2021 survey of 976 Masters weightlifters (499 females and 477 males, ages 35 to 88 years) from six countries participated in an online survey about injuries and training [11]. The cumulative incidence of polyarthralgia increased with age and was higher for post-menopausal females than males of similar age (*p<*0.01) (Fig. 2).

## Discussion

In this study we reported prevalence and determinants for arthralgia for different anatomical sites in 868 competitive female Masters weightlifters, ages 30 to 79, from 30 countries. The main findings were first, anatomical sites with a prevalence of more than 25% of arthralgia were knees, shoulders, hands/wrists, and hips; second, lifting heavier weights was associated with arthralgia but training frequency was not; third, menopausal status and more severe menopausal psychological symptoms were associated with arthralgia.

Arthralgia is a common occurrence and prevalence increases with age. Physical activity and resistance training is consistently recommended for managing joint pain, but there is a common perception that such activities may negatively impact the joints [7, 8]. However, arthralgia is not a contraindication to sport participation, and Masters weightlifters are an example. There are few studies on Masters athletes or about arthralgia in strength sports, especially among female sport participants [24]. This is important as there is a high sports participation rate for females, including in weightlifting [25]. To our knowledge, this is the first study on female Masters weightlifters who regularly train with heavy weights in a sport that put stress on multiple joints. Since females have a higher prevalence of arthralgia than males [26] and there is a possible connection with menopause [3], female weightlifters were the focus of this study.

### Prevalence of arthralgia in female weightlifters

Arthralgia was common among female weightlifters at all ages, and arthralgia increased with age, namely 41% at ages 35-44, 50% at ages 45-50, and 54% for respondents older than 60. The most common sites of joint pain were knees (39%), shoulders (30%), hands/wrists (29%), and hips (25%). It is important to note that the prevalence of joint pain at these anatomical sites was similar in community-dwelling females at approximately 58 years of age, drawn from clustered random samples in urban areas in Scotland [4]. Differences between weightlifters and this population were not statistically significant except for ankle joint pain which was lower in female weightlifters compared to the Scottish study (10% and 17%, respectively, *p<*0.01). There is a significant gap in the literature investigating female athletes. Prior studies often focused on OA in male athletes [2, 6, 27, 28], and many studies had small sample sizes [20]. The prevalence of OA was 31% in former male elite weightlifters in one study [6]. In a Finnish study of former elite male athletes degenerative disease at the lumbar spine was more common among weightlifters than among runners in former elite male athletes in a Finnish study at mean age 57 years [27]. However, training regimens may be more rigorous now with strength training included in all sports compared to what the Finnish athletes were exposed to.

### Risk factors for arthralgia in female weightlifters

Participation in prior strength sports (powerlifting, body building) was not a risk factor for arthralgia and could even be protective for shoulder joint pain. Participation in high impact sports and participation in competitions at a younger age were not associated with arthralgia in female weightlifters. This is in contrast to other studies where prior participation in high impact sports was a risk factor for OA [20].

Higher training load was a risk factor for arthralgia of the knees, hips, elbows, or hands. Prior injuries at the shoulders, spine, hips, knees, ankles were also risk factors for arthralgia at these joints. Studies have shown that joint-level factors such as injury, malalignment and abnormal loading of the joints are risk factors for arthralgia or OA [29, 30]. Repetitive stress on a joint or overuse increases the risk of arthralgia or OA at that joint [10]. On the other hand, joints adapt to increased use. Baker et al [31] concluded that regular participation in concurrent or cross training might alleviate the stress on joints and improve overall fitness and performance from responses in Masters swimmers. In weightlifting, maintaining muscle strength and conditioning could help prevent joint injury [32]. Prior to participating in any sport, it is important to consider body mass, previous joint injuries, occupational risk, and sport history to assess an individual’s risk of arthralgia [8]. Training programs that personalize intensity and frequency could be helpful in managing or preventing arthralgia.

### Arthralgia and menopause

Menopausal status was significantly associated with arthralgia. The proportion of athletes with arthralgia was 52% for pre-menopausal females, 62% for females undergoing natural menopause, and significantly higher with 73% in females undergoing medical or surgical menopause. To our knowledge differentiating between natural and medical or surgical menopause has not been considered before. The cumulative incidence of self-reported polyarthralgia increased in females at a faster rate after age 50 compared to males which could indicate a potential menopausal effect. Several studies have noted that joint pain or OA increases around the time of menopause [33–35], but sex differences could also be explained by factors such as bone loss or lack of muscle strength [30]. Since menopausal symptoms begin before the cessation of menses, it is important to consider whether there is a connection of arthralgia with severity of menopausal symptoms. In our study, severity of psychological menopausal symptoms (depressive mood, irritability, anxiety, and physical and mental exhaustion) was associated with arthralgia in all joints. This is aligned with results from a longitudinal study where depressed mood was shown to be associated with joint pain [19].

### Strengths and limitations

Strengths of this study were the large number of female weightlifters from 30 countries and considering determinants for arthralgia at different anatomical sites. There is a significant gap in the literature studying female athletes despite large participation rates. This was an online survey of joint pain in the last six months and prior injuries were self-reported and thus responses may suffer from recall bias. However, information on joint pain was collected through different questions such as own experience, visiting health professionals because of joint pain, or diagnosis by health professionals. The question about prior injuries in specific joints was accompanied by a question of at which age such an injury first occurred, but there was no information about the severity and duration of injuries. Study participants differed in years of experience and their sport history, thus those with longer exposure to the sport or participation in competitive sports at younger ages would be more likely to have experienced an injury or have cumulative stress on joints. Information on weekly hours of training and maximum weight achieved in the past six months was collected, but specific training load was not available. Future longitudinal studies in Masters weightlifters with more detailed information about training intensity would be useful to understand cumulative stress on specific joints and injuries requiring surgery or length of recovery until resumption of weightlifting or retirement from the sport.

## Conclusions

Age, BMI, and heavier weights lifted were associated with polyarthralgia. Prior strength training was protective of arthralgia in the shoulder joints, thus resistance exercises for isolated muscle groups may be beneficial. Menopausal status and severity of psychological menopausal symptoms (including depressive mood and anxiety) were associated with polyarthralgia. Athletes, coaches and sports medicine professionals should be aware that prevalence of polyarthralgia increases post-menopause, however it is not a contraindication to competitive weightlifting. Menopausal symptom severity needs to be considered in personalized training programs to avoid potential overtraining and possibly arthralgia. Strength exercises for isolated muscle groups may mitigate or protect against arthralgia. Individual weightlifting training programs should consider sport history and prior injuries to manage arthralgia.

## Data Availability

The datasets generated and/or analyzed during the current study are available in the Zenodo repository https://zenodo.org/deposit/7954380. https://doi.org/10.5281/zenodo.7954380.

## Abbreviations

OA: Osteoarthritis
MRS: Menopausal rating scale
IMWLA: International Masters Weightlifting Association
SHMF: Sinclair-Huebner-Meltzer-Faber points
